# Oxymetazoline hydrochloride ophthalmic solution, 0.1%, boosts the effects of botulinum toxin on blepharospasm: a case series

**DOI:** 10.1186/s13256-022-03493-6

**Published:** 2022-08-05

**Authors:** Jonathan Sung, Alice Song, Michael Song, Julia Song

**Affiliations:** 1 Southern California Eye Physicians & Surgeons, 1111 S. Fair Oaks Ave, Pasadena, CA 91105 USA; 2 Center for Oculofacial & Orbital Surgery, 10861 Cherry Street #208, Los Alamitos, CA 90720 USA

**Keywords:** Oxymetazoline hydrochloride ophthalmic solution (0.1%), Botulinum toxin, Blepharospasm, Blepharoptosis

## Abstract

**Background:**

Oxymetazoline hydrochloride ophthalmic solution (0.1%) is a medication used to treat blepharoptosis. Patients who suffer from blepharoptosis have low-lying eyelids that can hinder their vision. Oxymetazoline hydrochloride ophthalmic solution (0.1%) is prescribed to patients to improve their vision by lifting the upper eyelids. Blepharospasm consists of involuntary, bilateral orbicularis oculi muscle movements that result in twitching and eyelid closure. Botulinum toxin is a treatment used to treat blepharospasm by preventing muscle contraction; but it is not always effective.

**Case presentation:**

The effects of treatment with both oxymetazoline hydrochloride ophthalmic solution (0.1%) and botulinum toxin are assessed in three patients: (1) Patient A, a 58-year-old Filipina woman; (2) patient B, a 62-year-old Korean woman; and (3) patient C, A 57-year-old Vietnamese woman. All patients had been diagnosed with blepharoptosis as well as blepharospasm. Each patient was given an opportunity to complete an optional survey to assess not only the efficacy of oxymetazoline hydrochloride ophthalmic solution (0.1%) together with botulinum toxin but also their perceived stress during the past month.

**Conclusions:**

Administering botulinum toxin for the treatment of blepharospasm in patients A and B yielded the expected results; adding oxymetazoline hydrochloride ophthalmic solution (0.1%), a medical treatment for ptosis, to the treatment regimen yielded an unexpected reduction of blepharospasm. We propose that botulinum toxin and oxymetazoline hydrochloride ophthalmic solution (0.1%) can have a synergistic effect on reducing blepharospasm when used concomitantly. We present three cases in which combined use of botulinum toxin with oxymetazoline hydrochloride ophthalmic solution (0.1%) reduced blepharospasm, and propose possible reasons for such effects. We also discuss previous literature in agreement with the results of our cases.

## Introduction

Blepharospasm is an uncommon condition that consists of involuntary movements of the orbicularis oculi muscles [1]. Individuals who experience blepharospasm are usually female, typically between the ages of 50 and 70 years [2]. Blepharospasm solely targets the periocular region, affecting the orbicularis oculi muscles that are responsible for eyelid closure and movement. As a result of targeting a single location, blepharospasm is considered to be a focal dystonia [2]. Dystonia refers to the constant or irregular contractions of muscles as involuntary occurrences.

Blepharospasm can result from genetic and environmental factors [3]. The genes contributing to an increased risk of blepharospasm include* GNAL*,* CIZ1*,* TOR1A*,* DRD5*, and* REEP4* [2]. These genes code for proteins involved in muscle function, including the alpha G-protein subunit that influences the activity of adenylyl cyclase [2]. Overstimulation of adenylyl cyclase can cause an increase in the production of cyclic adenosine monophosphate (cAMP), leading to more frequent orbicularis oculi muscle contractions [2]. Environmental influences may consist of prolonged exposure to television, computers, and other activities causing eye strain. These activities may slightly increase the risk of developing blepharospasm [2].

Botulinum toxin works as an acetylcholine (ACh) antagonist to limit the activity of the muscles. By blocking the binding of ACh neurotransmitters at the postsynaptic receptors in the neuromuscular junction, botulinum toxin hinders the ability of muscles to contract [4]. Due to its ability to act as an antagonist by blocking contractions, botulinum toxin can treat muscle dystonias [4]. Botulinum toxin was initially approved to treat strabismus in 1989 by the U.S. Food and Drug Administration (FDA) and was approved to treat blepharospasm later in the same year [4].

Abnormally low-lying upper eyelids is a condition known as blepharoptosis [5]. Blepharoptosis can be classified as acquired, neurogenic, myogenic, mechanical, aponeurotic, or traumatic [5]. The severity of blepharoptosis can range from mild to severe, with mild blepharoptosis having less impact on the visual field than severe blepharoptosis [5].

Oxymetazoline hydrochloride ophthalmic solution (0.1%) (Upneeq®; RVL Pharmaceuticals, Inc., Bridgewater, NJ, USA) was recently approved by the FDA in 2020 to treat blepharoptosis in adults [6]. This ophthalmic solution (0.1%) works by increasing the activity of Müller's muscle α-adrenergic receptors, leading to stronger contractions in Müller's muscle [7], the normal function of which is to keep the upper eyelids elevated. Blepharoptosis may arise if Müller's muscle is damaged or weakened or if levator dehiscence is present [5].

A review of the literature demonstrates that botulinum toxin has been successfully used to treat blepharospasm, and that oxymetazoline hydrochloride ophthalmic solution (0.1%) has been successfully used to treat blepharoptosis. In a 2020 study that assessed the effectiveness of botulinum toxin on blepharospasm, a high-speed camera and micro-light-emitting diodes were used to analyze the movements of 78 eyelids [8]. The study generated a mathematical algorithm known as the Fast Fourier Transform (FFT) algorithm to quantify energy expenditure from these eye movements [8]. The authors of the study reported a significant reduction in the amount of energy expended from blepharospasm after the administration of botulinum toxin (*p* value = 0.0018) [8]. A 2019 study assessed the efficacy of oxymetazoline hydrochloride ophthalmic solution (0.1%) to reduce blepharoptosis [7]. The Leicester Peripheral Field Test (LPFT) was used to analyze the visual fields of participants before and after oxymetazoline hydrochloride ophthalmic solution (0.1%) use. A significant improvement in the results of the LPFTs was observed when eyelid height before and after oxymetazoline hydrochloride ophthalmic solution (0.1%) administration was compared (*p*-value < 0.001) [7]. The authors of the study concluded that oxymetazoline hydrochloride ophthalmic solution (0.1%) was effective in treating acquired blepharoptosis, a positive outlook for future treatments [7].

## Case presentation

We present three cases in which oxymetazoline hydrochloride ophthalmic solution (0.1%) was administered after botulinum toxin treatment. Patient A was a 58-year-old Filipina woman; patient B was a 62-year-old Korean woman; and patient C was a 57-year-old Vietnamese woman. All patients had been diagnosed with mechanical blepharoptosis and blepharospasm. Botulinum toxin was administered to treat the blepharospasm, and oxymetazoline hydrochloride ophthalmic solution (0.1%) was administered to treat the blepharoptosis. These cases were particularly very intriguing, as we observed in Patients A and B that oxymetazoline hydrochloride ophthalmic solution (0.1%) increased the effects of botulinum toxin and resulted in a greater reduction of blepharospasm. In Patient C, we observed the initial effects of oxymetazoline hydrochloride ophthalmic solution (0.1%) after 4 days and reported the preliminary results. Each patient was given the option to complete a survey that assessed the efficacy of botulinum toxin alone and the efficacy of both botulinum toxin and oxymetazoline hydrochloride ophthalmic solution (0.1%) in reducing blepharospasm. Furthermore, the survey asked patients about their perceived stress [9] during the past month. According to the Perceived Stress Scale questionnaire, [9] a score of 0-13 indicates low levels of psychological stress, a score of 14-26 indicates moderate stress, and a score of 27-40 indicates high stress.

**Fig. 1:**
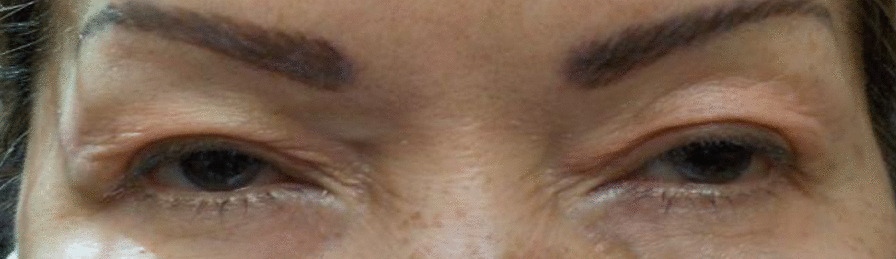
Patient A before using oxymetazoline hydrochloride ophthalmic solution (0.1%)

Patient A’s past medical history included hypertension and temporomandibular joint disorder. Her ocular history included blepharospasm, upper eyelid ptosis, upper lid dermatochalasis, and dry eye syndrome. She had been using brimonidine tartrate ophthalmic solution (0.15%), carvedilol 6.25 mg oral tablet, desvenlafaxine succinate 25 mg oral tablet, lorazepam 0.5 mg oral tablet, losartan potassium 25 mg oral tablet, and oxymetazoline hydrochloride ophthalmic solution (0.1%). Silicone punctal plugs had been inserted to treat her dry eye on 20 October 2016. She had been receiving botulinum toxin treatment since 2019. The administration of oxymetazoline hydrochloride ophthalmic solution (0.1%) to treat her blepharoptosis began on 17 March 2021 (Fig. 1).

**Fig. 2:**
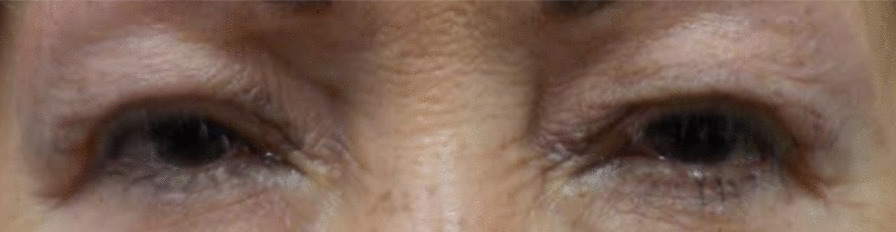
Patient A after using oxymetazoline hydrochloride ophthalmic solution (0.1%) for 4.5 months

Patient A’s most recent follow-up examination took place on 9 July 2021 at which time her visual acuities (Va) were 20/20 in the right eye (OD) and 20/20 in the left eye (OS). She had observed a noticeable decrease in her blepharospasm after oxymetazoline hydrochloride ophthalmic solution administration (0.1%). She spoke of not having to use as much energy to keep her eyes open while using the oxymetazoline hydrochloride ophthalmic solution (0.1%) treatment. Patient A received her 19th botulinum injection with nerve block on 9 July 2021 and continues to use the oxymetazoline hydrochloride ophthalmic solution (0.1%) in both eyes (OU) every morning. According to the survey, Patient A indicated that using oxymetazoline hydrochloride ophthalmic solution (0.1%) after a botulinum toxin administration was effective and resulted in little to no blepharospasm after 4.5 months of using oxymetazoline hydrochloride ophthalmic solution (0.1%) (Table 1) (Fig. 2). Patient A had a low stress score of 13 for the past month (Table 2).

**Table 1.** Results of the Leicester Peripheral Field Test Survey

Patient	Efficacy of botulinum toxin on its own	Efficacy of botulinum toxin with oxymetazoline hydrochloride ophthalmic solution (0.1%)
A	1	3
B	1	3
C	2	2

Efficacy scores: 0 = not effective with frequent spasm; 1 = slightly effective with moderate spasm; 2 = effective with little spasm; 3 = very effective with no spasm

**Table 2:** Scores of the Perceived Stress Scale questionnaire

Question	Patient A scores	Patient C scores
1. In the last month, how often have you been upset because of something that happened unexpectedly?	2	2
2. In the last month, how often have you felt that you were unable to control the important things in your life?	1	2
3. In the last month, how often have you felt nervous and stressed?	2	2
4. In the last month, how often have you felt confident about your ability to handle your personal problems?	1	2
5. In the last month, how often have you felt that things were going your way?	3	2
6. In the last month, how often have you found that you could not cope with all the things that you had to do?	1	2
7. In the last month, how often have you been able to control irritations in your life?	3	2
8. In the last month, how often have you felt that you were on top of things?	3	1
9. In the last month, how often have you been angered because of things that happened that were outside of your control?	1	2
10. In the last month, how often have you felt difficulties were piling up so high that you could not overcome them?	0	2
Total score^a^ (Reverse responses to #4, #5, #7, #8: 0 = 4, 1 = 3, 2 = 2, 3 = 1)	13	21

Scores: 0 = never; 1 = almost never; 2 = sometimes; 3 = fairly often; 4 = very often

Patient B deferred to complete this part of the study

^a^Total score: 0-13 indicates low levels of psychological stress, 14-26 indicates moderate stress, and 27-40 indicates high stress

Patient B’s past ocular history included blepharospasm, blepharoptosis, dry eyes, narrow-angle glaucoma, and cataract. She had been using Xiidra ophthalmic solution (Xiidra® 5%; Novartis, Copenhagen, Denmark) in OU twice daily (bid), Lumigan (latanoprost) OU every night (qhs), and oxymetazoline hydrochloride ophthalmic solution (0.1%) OU every morning (qam). Punctal silicone plugs had been inserted to treat her dry eye on 19 December 2016. Patient B had been receiving botulinum toxin treatment since 2016. The administration of oxymetazoline hydrochloride ophthalmic solution (0.1%) to treat Patient B’s blepharoptosis began on 10 June 2021.

**Fig. 3:**
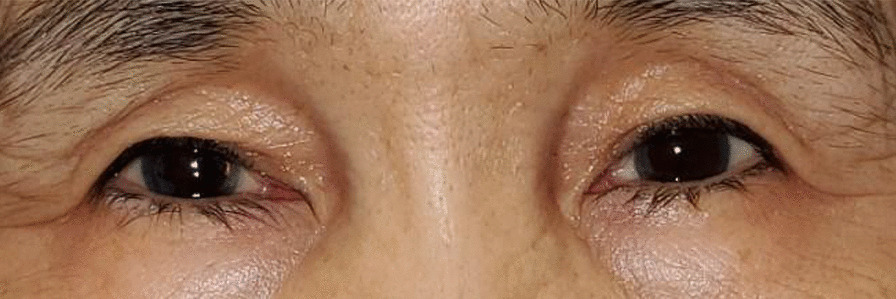
Patient B after using oxymetazoline hydrochloride ophthalmic solution (0.1%) for 6 months

Patient B’s most recent follow-up examination took place on 15 July 2021. Her Va was 20/80-1 OD and 20/20-3 OS. She reported a decrease in her blepharospasm when using oxymetazoline hydrochloride ophthalmic solution (0.1%). Her most recent botulinum toxin treatment was on 29 October 2021 as was her seventh botulinum treatment. Patient B continues to use oxymetazoline hydrochloride ophthalmic solution (0.1%) OU qam. According to the survey, patient B indicated that the use of oxymetazoline hydrochloride ophthalmic solution (0.1%) after botulinum toxin administration was effective and resulted in little to no blepharospasm after 1 month of its use (Table 1). Patient B deferred to complete the Perceived Stress Scale questionnaire. After 6 months of using oxymetazoline hydrochloride ophthalmic solution (0.1%), Patient B indicated a strong reduction in blepharospasm (Fig. 3) during her most recent examination.

Patient C’s past medical history included systemic lupus erythematosus. Her ocular history included blepharospasm, blepharoptosis, chalazion, dry eyes, and lagophthalmos. She had been using artificial tears and oxymetazoline hydrochloride ophthalmic solution (0.1%). She had been receiving botulinum toxin injections for blepharospasm since 2015.

**Fig. 4:**
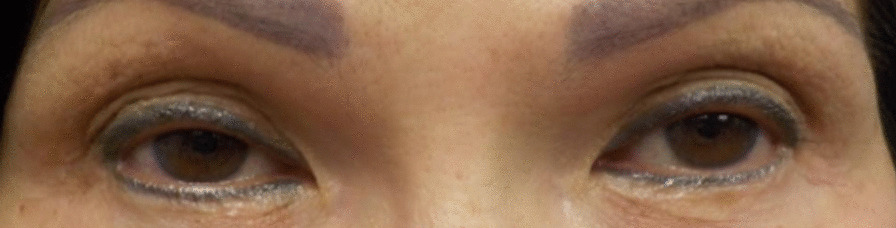
Patient C before using oxymetazoline hydrochloride ophthalmic solution (0.1%)

**Fig. 5:**
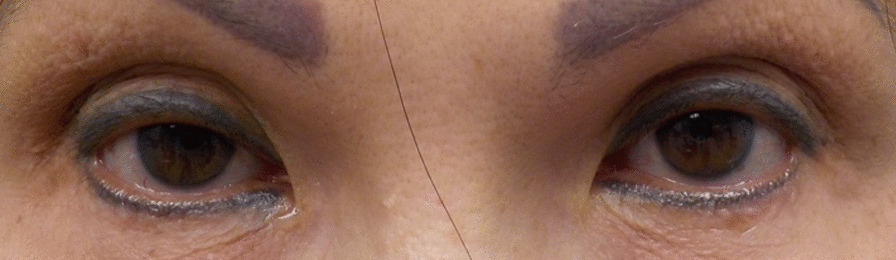
Patient C after using oxymetazoline hydrochloride ophthalmic solution (0.1%) for 6 months

On patient C’s most recent follow-up on 29 July 2021 at which time her VA was 20/20 OD and 20/20 OS. She had arrived for an administration of botulinum toxin to treat her blepharospasm. Patient C received her 34th botulinum toxin administration on 29 July 2021. Oxymetazoline hydrochloride ophthalmic solution (0.1%) was given to her after her examination as another treatment for blepharospasm. According to the survey, patient C indicated that oxymetazoline hydrochloride ophthalmic solution (0.1%) use after botulinum toxin administration did not result in a noticeable blepharospasm reduction after 4 days (Fig. 4). Patient C specified that only a small reduction in blepharospasm took place after 4 days of using oxymetazoline hydrochloride ophthalmic solution (0.1%), yet she decided to continue using oxymetazoline hydrochloride ophthalmic solution (0.1%) long-term to potentially experience more noticeable reductions (Table 1). She specified that both botulinum toxin treatment by itself and botulinum toxin treatment with oxymetazoline hydrochloride ophthalmic solution (0.1%) were effective with moderate spasm frequency. Patient C had a Perceived Stress Scale score of 21, indicating moderate stress, for the past month (Table 2). After 6 months of using oxymetazoline hydrochloride ophthalmic solution (0.1%), Patient C observed a strong reduction of blepharospasm, and her blepharoptosis improved (Fig. 5).

## Discussion and conclusion

There was a noticeable reduction in blepharospasm in patient A from the combination treatment of both oxymetazoline hydrochloride ophthalmic solution (0.1%) and botulinum toxin. She specified that botulinum toxin treatment by itself was not as effective as the combination of botulinum toxin and oxymetazoline hydrochloride ophthalmic solution (0.1%). She also stated that she will continue to use oxymetazoline hydrochloride ophthalmic solution (0.1%) for a long time, emphasizing that it has had a major positive impact on her life. Based on patient A’s Perceived Stress Scale score of 13, the high effectiveness of oxymetazoline hydrochloride ophthalmic solution (0.1%) and botulinum toxin together may correlate with lower levels of stress (Table 2). After using oxymetazoline hydrochloride ophthalmic solution (0.1%), patient A then required botulinum toxin administration every 3 months instead of every several weeks. She stated that her eyes opened more, and she observed a noticeable improvement in vision (Fig. 2).

Patient B also noticed a greater reduction in blepharospasm when using oxymetazoline hydrochloride ophthalmic solution (0.1%). Although she deferred to complete the Perceived Stress Scale score on the survey, she verbally expressed a noticeable reduction in blepharospasm (Table 1). The effects she experienced closely resembled the effects patient A experienced.

In contrast, patient C noticed only minimal reduction in blepharospasm after using oxymetazoline hydrochloride ophthalmic solution (0.1%) and botulinum toxin treatment together for 4 days. When patient C completed the survey, she had only been using oxymetazoline hydrochloride ophthalmic solution (0.1%) for 4 days, in comparison to the several months of use by the other two patients, a possible indication that noticeable reductions in blepharospasm may take longer to appear (Fig. 4). There was also a possibility that Müller's muscle in each patient responded differently to oxymetazoline hydrochloride ophthalmic solution (0.1%). Patient C experienced moderate levels of stress according to her Perceived Stress Scale score [9]. It is possible that higher stress levels may correlate with reducing the efficacy of oxymetazoline hydrochloride ophthalmic solution (0.1%) when paired with botulinum toxin (Table 1). Oxymetazoline hydrochloride ophthalmic solution (0.1%) may cause greater blepharospasm reductions over longer periods of time.

The precise biochemical mechanism for the effect of oxymetazoline hydrochloride ophthalmic solution (0.1%) on blepharospasm is still being researched, but some preliminary conclusions can be drawn. Because oxymetazoline hydrochloride ophthalmic solution (0.1%) stimulates contractions in Müller's muscle, and Müller's muscle is responsible for maintaining upper eyelid elevation, reductions in blepharospasm may have been due to the strengthening of Müller's muscle according to findings from an earlier study [5]. The results from another earlier study also suggested that the upper eyelids would remain elevated, preventing involuntary closure of the eyelids resulting from these spasms [7]. Furthermore, it is possible that the reduction in involuntary orbicularis muscle contraction with botulinum toxin and the stimulation of Müller's muscle with oxymetazoline hydrochloride ophthalmic solution (0.1%) synergistically reduced any irregular activity in the muscles surrounding the eyes [4, 6]. More studies need to be done on the potential relationship between Müller's muscle and the orbicularis oculi muscles associated with blepharospasm.

Our presentation of three cases involving a reduction in blepharospasm due to botulinum toxin and oxymetazoline hydrochloride ophthalmic solution (0.1%) demonstrated a unique phenomenon of synergism. Based on evidence from prior studies, both botulinum toxin and oxymetazoline hydrochloride ophthalmic solution (0.1%) are effective in treating blepharospasm and blepharoptosis, respectively. Our investigation of the effects of oxymetazoline hydrochloride ophthalmic solution (0.1%) brings new evidence to light, suggesting that oxymetazoline hydrochloride ophthalmic solution (0.1%) may help reduce blepharospasm when combined with botulinum toxin.

## Data Availability

Data and materials are available upon request. The datasets supporting the conclusions of this article are included within the article and its additional files.

## Abbreviations

Ach: Acetylcholine
bid: 2 Times a day
cAMP: Cyclic adenosine monophosphate
FDA: Food and Drug Administration
OD: Right eye
OS: Left eye
OU: Both eyes
qam: Every morning
qhs: Every night
Va: Visual acuity
